# Sharing social media data: The role of past experiences, attitudes, norms, and perceived behavioral control

**DOI:** 10.3389/fdata.2022.971974

**Published:** 2023-01-16

**Authors:** Esra Akdeniz, Kerrin Emilia Borschewski, Johannes Breuer, Yevhen Voronin

**Affiliations:** ^1^Data Services for the Social Sciences, GESIS - Leibniz Institute for the Social Sciences, Cologne, Germany; ^2^Survey Data Curation, GESIS - Leibniz Institute for the Social Sciences, Cologne, Germany; ^3^Center for Advanced Internet Studies (CAIS), GESIS - Leibniz Institute for the Social Sciences, Cologne, Germany

**Keywords:** social media data, Theory of Planned Behavior, data sharing, data reuse, data management

## Abstract

Social media data (SMD) have become an important data source in the social sciences. The purpose of this paper is to investigate the experiences and practices of researchers working with SMD in their research and gain insights into researchers' sharing behavior and influencing factors for their decisions. To achieve these aims, we conducted a survey study among researchers working with SMD. The questionnaire covered different topics related to accessing, (re)using, and sharing SMD. To examine attitudes toward data sharing, perceived subjective norms, and perceived behavioral control, we used questions based on the Theory of Planned Behavior (TPB). We employed a combination of qualitative and quantitative analyses. The results of the qualitative analysis show that the main reasons for not sharing SMD were that sharing was not considered or needed, as well as legal and ethical challenges. The quantitative analyses reveal that there are differences in the relative importance of past sharing and reuse experiences, experienced challenges, attitudes, subjective norms, and perceived behavioral control as predictors of future SMD sharing intentions, depending on the way the data should be shared (publicly, with restricted access, or upon personal request). Importantly, the TPB variables have predictive power for all types of SMD sharing.

## 1. Introduction

As in other scientific disciplines conducting empirical research, in the social sciences, research data constitute a cornerstone of scientific knowledge. For many decades, the most widely used type of data in the quantitative social sciences has been survey data. In recent years, however, other forms of data have become increasingly important for gaining novel insights into human behavior (Ledford, 2020). One type of data that has recently seen a particular increase in use in the social sciences is social media data (SMD)^1^. SMD not only offer the possibility to study a broad range of topics but also allow the investigation of research questions with large samples that can be monitored continuously (and with high temporal resolution) over long periods of time and at much lower costs compared to survey data (van Atteveldt and Peng, 2018; Hagen et al., 2019a). The trend to conduct research with SMD is reflected in the increasing number of publications that use this type of data. This is a clear indicator that social media are not only interesting subjects of study but also increasingly important data sources in the social sciences (Weller and Kinder-Kurlanda, 2015; van Atteveldt and Peng, 2018; Breuer et al., 2021b).

Importantly, one precondition for research with SMD is that researchers can access them. For other types of data, there are essentially two general access paths: researchers can either collect the data themselves (primary data use) or reuse^2^ previously collected data (secondary data use). Notably, access to SMD is associated with new challenges that are different from those, e.g., for survey data (Breuer et al., 2020; Leonelli et al., 2021). These challenges are important to consider and address, as they might negatively affect data quality or even prevent researchers from working with SMD. The challenges associated with accessing SMD fall into different categories and, e.g., concerns include required technical expertise or legal regulations. In addition to legal and technical obstacles that can make sharing social media data difficult, there is a notable lack of standards for the documentation as well as established procedures and technical solutions for archiving and disseminating social media data. This is, e.g., different for more traditional types of data, such as survey data. This example shows that, while some of the challenges are the same for SMD and other types of data (e.g., the time and effort it takes to properly document them), others are unique or at least more pronounced for this data type (e.g., restrictions in data access or the lack of documentation standards). One potential consequence of these challenges and differences in resources for addressing them is an increased inequality in terms of data access and a divide between the “data haves” and the “data have nots,” as Weller and Kinder-Kurlanda (2015, *p*. 31) put it. For those reasons, it is even more important that researchers do not keep the data to themselves once they have been collected. Sharing^3^ the data with other researchers can reduce and prevent the inequality in data access. Apart from this, there are many other reasons for sharing and reusing data in general (regardless of the data type). According to the European Commission (2016, 2017) and the National Science Foundation (2020), data sharing is a matter of ethics in research and a duty to the scholarly community, the public, and other researchers. It helps in making research transparent, increases efficiency (as data do not have to be collected multiple times for the same purpose), enables reproducibility, and increases replicability. Data sharing is also a way to combat questionable research practices and improve the quality of peer review (Abele-Brehm et al., 2019).

Adhering to the FAIR principles^4^, according to which data should be findable, accessible, interoperable, and reusable, creating data management plans and using metadata standards facilitate the management and sharing of data (Wilkinson et al., 2016). Given the importance of data for empirical research, some have even argued that data sharing is a professional responsibility for researchers that can also be seen as a moral obligation (Borgman, 2010; Kim, 2013; Hagen et al., 2019b). Across scientific disciplines, open science practices are becoming increasingly common, and publishers, funders, and other stakeholders are increasingly demanding structured data management and access to research data as a prerequisite for funding and publishing (European Commission, 2016, 2017).

This already illustrates that the reasons for sharing research data can be different. In addition to requirements from funders and publishers, researchers may also be motivated by altruistic motives, such as contributing to the academic community or leading by example to promote values of transparency. Importantly, sharing data also has benefits for researchers who do so (Kim and Stanton, 2012; Jeng et al., 2016). Even though sharing data requires some additional work and resources, it can have significant benefits for researchers' careers in the long run (Kling and Spector, 2002; Kankanhalli et al., 2005; Borgman, 2010; Kim and Stanton, 2012; Kim, 2013, 2017; Altman, 2016; Toribio-Flórez et al., 2020). For example, studies have shown that articles with shared research data have higher citation rates and, hence, a greater impact (Piwowar et al., 2007; Drachen et al., 2016; Park and Wolfram, 2017). From a practical perspective, creating proper documentation for data sharing not only saves time and resources for researchers who reuse the shared data but also for the researchers who created the data, as it facilitates revisiting old projects and reusing data and analysis code later on (Kim and Stanton, 2012; Kim, 2013; Houtkoop et al., 2018; Van Atteveldt et al., 2019).

Despite the benefits it entails, sharing research data is still not a widely established practice in the social sciences. This issue is even more pronounced for SMD. Weller and Kinder-Kurlanda (2015) found in their qualitative study that it was common for social media researchers to share their data within their research group, however, the data were rarely shared publicly. Instead, the data were often shared using unofficial channels, e.g., relying on personal contacts. While quite a few studies have investigated why researchers in the social and behavioral sciences and other disciplines do or do not share their data (Kim and Stanton, 2012; Kim, 2013; Houtkoop et al., 2018; Van Atteveldt et al., 2019), the literature on SMD sharing behavior is scarce. We will discuss both lines of research (i.e., on data sharing in general and SMD in particular) in the following section. Taking into account previous findings on data sharing practices as well as existing considerations and insights regarding SMD, our paper has two main aims: (1) Investigating the experiences and practices of researchers working with SMD and (2) Gaining insights into researchers' sharing behavior and influencing factors for their decisions. To achieve these aims, we conducted a survey study among social scientists working with SMD.

The theoretical framework for our empirical investigation of factors affecting the sharing of social media data is the Theory of Planned Behavior (TPB). TPB, posits that a behavioral intention is preceded by three influencing factors: norms, attitudes, and perceived existing capacities. In previous studies, the TPB has proven to be a useful theory for explaining data sharing (e.g., Kim and Stanton, 2012; Zenk-Möltgen et al., 2018). While previous studies have looked at types of data traditionally used in the social sciences (mainly survey data), this is the first study to focus on and apply the lens of TPB to the sharing of social media data. While there certainly are similarities in the decision process between, e.g., SMD and survey data, SMD have some specific attributes that can complicate data sharing. Key aspects in this context relate to perceived capacities (e.g., a lack of technical resources), but also attitudes, such as the fear of violating legal regulations or ethical guidelines. At the same time, with the growth of open science and different initiatives for promoting the transparency of research, researchers likely feel increasing social pressure to share data with the community. In general, we can distinguish between individual and systematic factors that affect data sharing behavior. Systematic factors, e.g., include the existence of infrastructures, requirements by funders, publishers, etc., and the general “data sharing culture” within disciplines/fields. Individual factors relate to the experiences, motivations, and expectations of individual researchers. The focus of TPB is mainly on such individual-level factors. Of course, TPB is not the only theoretical framework that can be applied to study factors influencing data sharing decisions on the individual level. For example, social exchange theory (Dillman, 1978) and the theory of contextual integrity (Nissenbaum, 2018) have been used to explain why study participants may or may not share their data with researchers. As the name indicates, social exchange theory focuses on data sharing as an exchange and, hence, stresses the aspect of trust. The theory of contextual integrity focuses more on situational parameters, such as the recipient of the data and the transmission principles. While these theories can also be used to investigate the sharing of SMD by researchers, they typically relate to specific cases or scenarios. The strengths of TPB are (a) that it can be used to assess general attitudes and intentions and (b) that it can capture multiple relevant dimensions on the individual level, including attitudes, perceived norms, and capacities.

For those reasons, in our study, we employ a TPB lens to investigate whether or to what degree past experiences, individual attitudes, disciplinary norms, and personal capacities in the sense of perceived behavioral control determine data sharing behavior for SMD, distinguishing between three data sharing forms. As SMD (as well as other types of research data) can be shared in different ways, we will also assess whether the relevance of motivators and barriers differs across three common data sharing modes: sharing data upon personal request, publicly without any restrictions and under controlled access.

### 1.1. Barriers to sharing research data

Several studies have investigated why researchers may be hesitant to share their data. This literature has identified several reasons. Most of those concern all types of research data. Notably, however, many of those reasons are especially pronounced for SMD, and some are even unique to them. In the following, we briefly discuss nine key reasons^5^ that have been identified in previous research, focusing on those that are either unique to or especially pronounced for SMD.

#### 1.1.1. Reason 1: Preparing data for sharing is resource-intensive

The process of data sharing and documentation takes up resources, and some researchers find this work too burdensome and time-consuming (Hemphill et al., 2021). Instead, researchers tend to prefer using most of their resources and time for the processes of data acquisition and their data analysis as well as writing publications based on their results (Sayogo and Pardo, 2013; Lane et al., 2014; Tenopir et al., 2015; Weller and Kinder-Kurlanda, 2015; Zenk-Möltgen et al., 2018; Van Atteveldt et al., 2019; Hemphill et al., 2021; Thoegersen and Borlund, 2022). Taking into account the particular format of this data type as well as the lack of documentation standards, this issue is even more pronounced for SMD.

#### 1.1.2. Reason 2: Not enough credit for data sharing

Currently, there are neither standards nor established community practices for data citation in most fields. Many researchers worry that they will not get credit for publishing data and sometimes also fear that they might lose ownership and “first rights” on the data when sharing them. Hence, they may prefer to abstain from sharing their data (Sieber and Trumbo, 1995; Moss et al., 2015; Park and Wolfram, 2017; Hemphill et al., 2021). Considering the amount of effort that is required for SMD sharing (see above), many researchers may be hesitant due to the discrepancy between this effort and the outcome/benefits for themselves (or their career).

#### 1.1.3. Reason 3: Lack of confidence and knowledge

Another reason why researchers may be hesitant to share their data is a lack of knowledge or confidence regarding the process (Acord and Harley, 2013). More specifically, researchers often feel that they do not have the knowledge needed to prepare the data. Further barriers might be a lack of knowledge about existing infrastructures or a lack of clarity about how the data sharing process works in practice (Zenk-Möltgen et al., 2018; Toribio-Flórez et al., 2020; Thoegersen and Borlund, 2022). The lack of knowledge on data preparation, documentation, and options for archiving data is even more pronounced for SMD, where researchers often feel “insecurity, uncertainty and aggravation” (Weller and Kinder-Kurlanda, 2015, *p*. 36) in regard to data sharing.

#### 1.1.4. Reason 4: Data protection laws

Another challenge concerns data protection laws. For example, in the European Union, the use of research data must conform to the General Data Protection Regulation (GDPR) (Breuer et al., 2020; Leonelli et al., 2021). It is especially challenging to anonymize SMD, and the process is much more complex compared to survey data. SMD need (sometimes intensive) processing before they can be shared. Hence, anonymization demands a substantial amount of resources, and even then, access might have to be restricted (Breuer et al., 2020; Sloan et al., 2020). Once the data are anonymized in a way that makes it impossible to identify individuals, they are likely to become less useful for further analysis (Thomson and Kilbride, 2015; Weller and Kinder-Kurlanda, 2015, 2016; Mannheimer and Hull, 2017; Sloan et al., 2020). The issue of privacy protection has been found to be among the most common reasons that prevent researchers from sharing their data in general as well as for SMD in particular (Borgman, 2012; Dehnhard et al., 2013; Fecher et al., 2015; Vanpaemel et al., 2015; Houtkoop et al., 2018; Zenk-Möltgen et al., 2018; Breuer et al., 2020; Hemphill et al., 2021).

In addition to aspects mentioned above, GDPR (2016) Article 5.1 imposes a certain purpose restriction, namely that “gathering of personal data is bound to specified, explicit and legitimate purpose”. Depending on the data and the way researchers intend to share them, this can be another obstacle for data re-use and sharing.

#### 1.1.5. Reason 5: Platform terms of service

A challenge that is specific to SMD is how to deal with the Terms of Services (ToS) of social media platforms and/or their application programming interfaces (APIs). If researchers collect data from social media platforms and use their APIs, they must follow the regulations laid out in their respective ToS to avoid losing data access (Mannheimer and Hull, 2017; Breuer et al., 2020, 2021a; Sloan et al., 2020; Assenmacher et al., 2021; Hemphill et al., 2021; Leonelli et al., 2021). Unlike legal regulations, such as the GDPR, the limitations for data sharing set in place by the companies and platforms have not only been established for reasons of data protection but also to protect their commercial interests (Lane et al., 2014; Thomson and Kilbride, 2015; Thomson, 2016; Hagen et al., 2019a,b; Assenmacher et al., 2021).

#### 1.1.6. Reason 6: Copyright

Another legal issue that also needs to be considered when sharing SMD is copyright. Since many public posts in the news feeds of social media users include images or content from third parties, such as media outlets or companies, they are likely protected by copyright. Copyright especially makes the sharing of raw data difficult (Hagen et al., 2019b; Breuer et al., 2021a).

#### 1.1.7. Reason 7: Informed consent

An issue that touches the legal as well as the ethical realm is that of informed consent. With SMD, it is often difficult to obtain informed consent from research subjects, especially if datasets have been obtained *via* APIs and contain a large number of subjects. There is a discussion in the community if agreeing to the general terms and conditions of the different platforms and services suffices and can be considered informed consent. However, because many social media users do not (thoroughly) read the general terms and conditions and probably did not consciously agree to the contents of the privacy policy, the dominant view is that agreement with platform ToS cannot be considered informed consent (Weller and Kinder-Kurlanda, 2015).

#### 1.1.8. Reason 8: Ethical challenges

Further factors that prohibit researchers from sharing their SMD concern ethical aspects. Researchers have concerns that they might be sharing sensitive data and violate the users' privacy (also unintentionally), even if they anonymize the dataset and take into account all legal requirements (Weller and Kinder-Kurlanda, 2015; Van Atteveldt et al., 2019). Guidelines for the ethical sharing and preservation of SMD have only recently started to emerge (Bishop and Gray, 2017; Weller and Kinder-Kurlanda, 2017; Hemphill et al., 2021), and the challenges differ vastly between platforms and types of data. In addition, Institutional Review Boards (IRBs) or ethics committees can make sharing SMD difficult or even impossible. IRBs may set regulations concerning privacy or data exchange that influence the reusability and shareability of the collected data (Thomson and Kilbride, 2015; Thomson, 2016; Assenmacher et al., 2021).

#### 1.1.9. Reason 9: Lack of common standards

The specific nature of SMD also requires specific forms of documentation. In general, there are no established and shared standards for the handling of SMD. This concerns issues of documentation (metadata) as well as those related to processing (e.g., anonymization). The need to develop such standards for SMD has existed for some time, but proposals for solutions have only started to emerge fairly recently (Weller and Kinder-Kurlanda, 2015; Thomson, 2016; Hagen et al., 2019b; Breuer et al., 2021b; Hemphill et al., 2021).

Among other reasons, prior studies have pointed to further aspects that are neither unique nor especially pronounced for SMD, such as fear of getting scooped (Savage and Vickers, 2009; Tenopir et al., 2011; Kim and Stanton, 2012; Campbell et al., 2019; Van Atteveldt et al., 2019; Hemphill et al., 2021; Thoegersen and Borlund, 2022), fear of misuse, misinterpretation, revelation of errors (Acord and Harley, 2013; Campbell et al., 2019; Van Atteveldt et al., 2019; Hemphill et al., 2021), and the uncertainty about the value of the data (Weller and Kinder-Kurlanda, 2015; Thoegersen and Borlund, 2022).

As we can see from this list of barriers and challenges, there are many reasons why researchers might not (want to) share their SMD. Even if they have a positive attitude toward data sharing, it seems plausible that the efforts (such as workload, time, costs) and perceived risks often outweigh the benefits that data sharing provides (Borgman, 2010; Tenopir et al., 2011; Acord and Harley, 2013; Fecher et al., 2015; Kim and Zhang, 2015; Abele-Brehm et al., 2019).

Despite this list of reasons and studies that illustrate the importance of data sharing as a subject of study, there are still many open questions regarding the sharing and reuse of SMD. Until now, there has been no systematic quantitative investigation of data sharing practices among researchers using social media data. Specifically, there is no quantitative research examining researchers' attitudinal, normative, behavioral beliefs, and past experiences that may influence their intentions to share SMD. With this study, we aim to address this. One key challenge for our study was to find a good balance between addressing and capturing general aspects related to sharing SMD and investigating specific factors that likely influence the data sharing decisions of individual researchers. This is not only important for properly operationalizing what we want to assess and formulating appropriate questions, but also for formulating recommendations based on our findings. The first step on the path from general considerations to specific insights that can form the basis for practical recommendations is the formulation of research questions that can be answered *via* our chosen method of a survey among researchers.

In the following sections, we will first present the research questions and the methods we employed for answering our research questions. We then describe the data analysis and the results of the survey. In the discussion section, we interpret and elaborate on the results. Finally, in the conclusion section, we provide a summary and also address limitations of the study as well as implications for future research.

## 2. Research questions

As discussed in the previous sections, there are many different factors that can influence decisions regarding the sharing of research data in general, and SMD in particular. Our focus in this study is on individual-level factors and we use the TPB as our theoretical framework for identifying, categorizing, and investigating these factors. Following this approach and considering the determinants that have been identified and studied in previous work, we cover four dimensions that we view as important for individual decisions regarding SMD: (1) experiences with data sharing and reasons for past data sharing decisions, (2) the role of attitudes, norms, and perceived behavioral control for future data sharing intentions, (3) the role of past experiences for future data sharing intentions, and (4) differences between different types of data sharing (publicly, under controlled access, or upon personal request).

### 2.1. Researchers' past behavior: Experiences and reasons for past data sharing decisions

The first aim of the study is to describe researchers' experiences with data sharing and to identify factors that may foster or hinder SMD sharing. Identifying these factors, as experienced by the researchers who are active in this domain, can be helpful for different stakeholders, such as funders, data archives, or publishers, for efforts to facilitate the process of sharing by developing services, guidelines, and recommendations. Hence, the first research question we seek to answer with our study is as follows:

RQ1: *What are researchers' past experiences, motivations, and barriers in regard to sharing SMD?*

### 2.2. Researchers' future behavior: The role of attitudes, norms, and perceived behavioral control in data sharing intentions

In addition to investigating past behavior and reasons for past SMD sharing decisions, our study also aims to investigate factors that predict future intentions in this regard. We do this through the lens of the Theory of Planned Behavior (TPB) by Ajzen (1991). According to the TPB, the best predictor of human behavior is behavioral intention, which is defined as a person's readiness to perform a specific behavior. We consider TPB as a relevant and helpful theoretical approach for investigating intentions regarding data sharing because data sharing decisions can be considered rational decisions as they are neither spontaneous behavior nor habits/habitual responses. Instead, data sharing can be considered as a form of planned behavior, preceded by rational considerations.

According to TPB, the intention to perform a behavior is determined by three central constructs:

- *Attitude toward the behavior*—defines how a person evaluates the behavior in general,
- *Subjective norm*—defines the role of perceived social pressure/expectations of others to show a specific behavior, and
- *Perceived behavioral control*—defines a person's perceptions about the autonomy and capability to show a specific behavior.

The TPB has successfully been used to explain a large variety of behaviors, for example, in the domain of health (Eves et al., 2003; Norman and Conner, 2006; Collins and Carey, 2007) but also in other areas, such as economic or environmental behavior (Heath and Gifford, 2002; Bamberg et al., 2003; Harding et al., 2007; Peng et al., 2014). The TPB has also been used to study the sharing of survey data (e.g., Kim and Stanton, 2012; Zenk-Möltgen et al., 2018).

Within this framework, it can be assumed that attitudes toward data sharing, perceived social pressure or approval, and perceived behavioral control can influence researchers' data sharing intentions. For example, researchers can be expected to be more likely to consider sharing their data with others if they have a positive attitude toward data sharing. On the other hand, institutional pressure regarding data sharing may increase due to requirements from funders or publishers or the increased prevalence of data sharing practices within a scientific community. Perceiving such pressures and expectations (by peers, funders, publishers, etc.) can influence researchers' subjective norms regarding data sharing.

Following the assumptions of the TPB, the perceived controllability of the behavior can also affect data sharing behavior. Perceived controllability is similar to the construct of self-efficacy proposed by Bandura (1994, p. 3), which reflects “individuals' confidence in their ability to organize and execute a course of action.” According to this view, peoples' motivation to show a particular behavior is based on what they believe they can do rather than their actual ability. Against the background of the assumptions proposed by the TPB regarding the role of attitudes, norms, and perceived behavioral control, the second research question for our study is as follows:

RQ2: *In what way do researchers' internal attitudes, external social norms, and perceived behavioral control predict future SMD sharing intentions?*

### 2.3. Researchers' future behavior: The role of past experiences in data sharing intentions

The TPB also points to possible background factors that can influence people's beliefs, such as previous experiences and behavior. The theory asserts that past behavior can be a predictor of future behavior. Different findings have already confirmed the relevance of past behavior as a predictor of intentions (Ouellette and Wood, 1998; Rhodes and Courneya, 2003; Knussen et al., 2004). In this study, three dimensions of past experiences are included: past data sharing, past data reuse, and experienced challenges.

For the specific case of sharing survey data, Zenk-Möltgen et al. (2018) investigated if researchers' past data sharing behavior is related to an increased intention to share data in the future. To this end, they analyzed correlations between the reported number of shared and cited research data or syntax codes and the frequency with which respondents said they had shared data and syntax codes over the last 3 years. The analysis showed a strong positive correlation between past data sharing and the intention to share survey data. Hence, it can be assumed that this is also the case for SMD.

This informs our third research question:

RQ3: *In what way(s) are researchers' past experiences SMD predictive of future data sharing intentions?*

### 2.4. Differences between different ways of sharing SMD

Similar to other research data, there are different ways in which SMD can be shared. While there are many more nuanced differences between actual sharing options that, e.g., depend on the functionalities of the platform or channels that are used for sharing, we broadly distinguish between three types of data sharing. First, the data can be shared completely openly without any restrictions. We label this public sharing. Second, researchers can employ various access restrictions. This is typically done *via* (curated) data archives. The kind and granularity of access control depends on what these archives offer, but some general options for access control include the use of embargoes, restrictive licenses, or an authorization process for users who want to reuse the data. Third, researchers may choose to only share data with selected researchers upon personal request. Notably, these sharing options not only differ from the perspective of the data reusers but also have different pros and cons for the researchers who share their data. Accordingly, the importance of the predictors for data sharing intentions identified in the previous sections (attitudes, norms, behavioral control, and past experiences) may also differ between sharing options. This leads to our fourth research question:

RQ4: *To what extent do the roles of various predictors of future SMD sharing intentions differ between data sharing options?*

## 3. Methodology

To answer the research questions presented above, we designed and conducted an online survey among researchers who work with SMD in which we asked about their experiences, attitudes, norms, perceived behavioral control, and intentions with regard to sharing SMD (Akdeniz et al., 2022). For simplicity, we use the term “researcher” to refer to all respondents in our study, which, in this case, are defined as follows: authors who have worked empirically with social media data and published articles in one of our selected journals. Notably, these authors may be academic researchers, but may also belong to other professional groups. The questionnaire covered different topics related to access to, (re)use, and sharing of SMD. In addition to those measures, questions about the demography (e.g., gender, age, current career stage) were included to obtain additional information about respondents and use them as control variables in our analyses. In the following, we will describe the recruitment process as well as the survey measures that we used to answer the research questions presented above. The full questionnaire can be found at Akdeniz et al. (2022). The online survey was administered *via* EFS Survey Unipark and was fielded from July to October 2021.

### 3.1. Recruitment process

The target population for this study was researchers who used SMD for their research and published journal articles based on SMD between 2018 and 2021. To recruit participants from this target group, e-mail addresses from authors of selected journal papers were gathered and included in the sampling frame. For this approach, two datasets were created, covering two levels: the journal level and the article level nested within journals. For the first level, information about journals was gathered by desktop research, inspecting top-ranked journals covering topics on social media, new data types, communication, and social networks according to the information and rankings by the Web of Science (Clarivate, 2022).^6^ 28 journals were selected. All of them published articles in English. Eight of them were golden open access journals, whereas 20 used hybrid access mode with subscription models but also (additional) options for open access publication. The list of journals that showed up in the results was inspected by the authors, and those journals that fulfilled the selection criteria outlined above were included as part of the sampling frame.^7^ The lower-level dataset, which contained articles, is nested in all the issues from January 2018 to April 2021 of the selected journals. The articles that qualified for the study were empirical articles (qualitative and quantitative) that used SMD. Examples of social media platforms that we considered relevant for our sampling procedure include Facebook, Twitter, Instagram, Wikipedia, Reddit, Tumblr, Twitch, WhatsApp, Telegram, Signal, TikTok, YouTube, and blogs. Online dating apps, webcam platforms, music apps, and podcasts did not qualify as SMD for our purposes. Articles excluded from the sampling process were those that were non-empirical (e.g., methodological, or theoretical papers about social media), interviews with (non) users of social media (incl. bloggers and live streamers), studies that used social media only as stimulus materials (e.g., for experiments) or exclusively used survey data or interview data (e.g., self-reported behavior or preferences regarding social media). For each article that qualified, we collected the authors' contact information [names, e-mail addresses, and their role for the article (1 = corresponding author, 2 = second author, etc.)]. These were used to recruit the participants for the survey. In the cases where an author's e-mail was not indicated in the publication, relevant e-mail addresses were gathered from institutional or personal webpages, from social media accounts, Open Researcher and Contributor ID (ORCID) accounts, or other scientific publications. The e-mail addresses were used to contact the authors. An initial invitation and a total of four reminders were sent to the researchers who were invited to participate between July and August 2021. Participation in the survey was voluntary^8^ and not compensated, and informed consent was obtained from all participants in the study prior to starting the online survey.

### 3.2. Participants

Overall, 1,738 authors were contacted, of which 253 responded (response rate: 20%). Four of the respondents reported that they had not used social media data for their research and were therefore excluded from the analysis. Accordingly, the results of the survey are based on the replies of the 249 respondents who had used SMD in their research.

Within the final sample of respondents, most were aged 31–40 (42.57%), 91.16% of the respondents were employed by a university, college, or technical university, and most were active in communication science (65.86%) and held a position as professor/assistant professor/associate professor (51%). The sample contained slightly more men (51.41%) than women (43.78%) (a complete table of the sample demographics can be found in Appendix B in Supplementary material).

### 3.3. Measures

The relevant concepts and the operationalization of the items^9^ are based on findings from prior studies on data sharing behavior in general (Borgman, 2012; Kim and Stanton, 2012; Dehnhard et al., 2013; Fecher et al., 2015; Vanpaemel et al., 2015; Houtkoop et al., 2018; Mannheimer et al., 2019) and SMD in particular (Thomson and Kilbride, 2015; Littman et al., 2018; Van Atteveldt et al., 2019; Breuer et al., 2020; Hemphill et al., 2021) as well as the TPB literature (Ajzen, 2006).

#### 3.3.1. Past experiences, motivators, obstacles, and challenges for data sharing

To assess past experiences, the survey included questions about past data sharing behavior, asking respondents whether they shared social media data with others outside of their research team.

To identify factors that may foster or hinder the sharing of SMD, the questionnaire contained two open-ended questions asking respondents about the reasons why they have or have not shared the SMD they collected for their research. These open-ended questions gave researchers the opportunity to elaborate on their reasons without limiting or influencing them with predefined answers.

Furthermore, a question on ethical and legal challenges was included based on previous findings indicating that the main challenges and reasons for researchers' hesitancy to share their data are concerns regarding legal regulations and ethics. The respondents were asked (*via* a multiple-choice question) which of the provided lists of legal or ethical challenges they faced when sharing or considering sharing social media data with others outside of their research team. The list of possible challenges was as follows:

- The Terms of Service of the data source do not permit sharing the data.
- Legal regulations differ across countries in collaborative projects.
- Uncertainties regarding legal regulations.
- The information investigated was sensitive.
- The research subjects were from a vulnerable population.
- Concerns regarding the privacy of participants.
- Other (please specify).
- None.

#### 3.3.2. Attitudes, norms, and perceived behavioral control related to SMD sharing

The wording of the items assessing attitude, norms, and control was based on the TACT framework: target, action, context, and time (Ajzen, 2006; Fishbein and Ajzen, 2010). An example item is “Generally speaking, sharing (*Action*) SMD (*Context*) with others outside of your research team (*Target*) within the next 3 years (*Time*), would be…”

The items were formulated for attitudes toward the behavior, subjective norms, and perceived behavioral control.

1. Attitude toward the behavior: Subjects indicated, in the sense of a global judgment, on a seven-point bipolar adjective scale whether they rate data sharing as valuable or worthless.
2. Subjective norm: Subjects indicated their level of agreement with statements on whether relevant referents, such as peers, would approve or not approve data sharing (other's expectation).
3. Perceived behavioral control: Subjects reported their level of agreement with statements indicating whether they feel capable of sharing the data and whether it is up to them to share the data (indicating the capability and autonomy of the researcher).

#### 3.3.3. Future data sharing intentions

As stated above, the key variable of interest for our research was future data sharing intentions. The operationalization of the data sharing intention was also defined according to TACT, and respondents were asked to provide their answers on a seven-point scale (1 = extremely unlikely, 2 = unlikely, 3 = somewhat unlikely, 4 = neither likely nor unlikely, 5 = somewhat likely, 6 = likely, 7 = extremely likely, with the additional option of “Don't know”) whether it is likely or unlikely that they will share SMD with others outside their research team within the next 3 years. This was asked for sharing SMD:

a. Publicly (with no restrictions),
b. Under controlled access (that regulates if and how data may be used by others),
c. Upon personal request (when being contacted directly by others).

## 4. Data analysis and results

**Figure 1:**
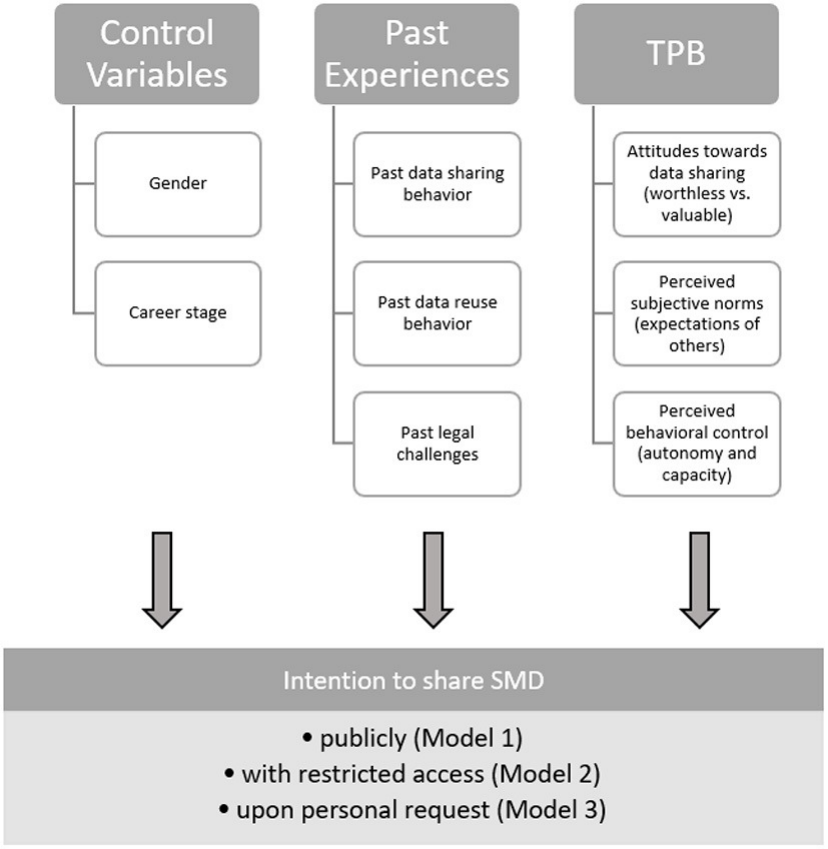
Theoretical model for our study.

To answer our first research question regarding the experiences and reasons for past SMD reuse and sharing decisions, we employed qualitative and descriptive quantitative analyses. To answer our second and third research questions about the predictors of future data sharing intentions, as well as our fourth research question asking about differences between data sharing options, we employed hierarchical (blockwise) linear regression models with measures of past experiences, attitudes, subjective norms, and perceived behavioral control as predictors and intentions for the different sharing options as outcomes. Data analysis was performed using the Stata 17 statistical software package (StataCorp, 2021). The correlation matrix of the variables used in the regression analysis can be found in Appendix B in Supplementary material. The structure and content of these models are summarized in Figure 1.

### 4.1. Researchers' past sharing behavior: Experiences, motivators, and obstacles

To answer the first research question (RQ1: *What are researchers' past experiences, motivations, and barriers in regard to sharing SMD?*), the respondents of the survey were asked several closed and open-ended questions about their experiences with data sharing. A total of 244 (98%) respondents had collected SMD themselves (primary use) before (multiple responses were possible, *N* = 249), whereas only 84 respondents had previously engaged in secondary use of SMD (33.73%). Respondents were also asked open-ended questions about their reasons for sharing or not sharing their SMD. The open-ended questions were analyzed, and a categorization scheme was developed to summarize the provided answers into coherent categories. Categories were derived manually in a bottom-up manner based on the data (*ad-hoc* coding) by one researcher and were checked by multiple coders. In the case of a difference of opinion among them, these were discussed and final decisions were made by the majority. We employed an a theoretical explorative and fully data-driven approach for the development of the coding categories as we had no a priori expectations regarding the types of answers that may be provided.

The results^10^ show that three main motives emerged for sharing SMD (open question, multiple responses possible, *N* = 94): On the one hand, data are shared for idealistic or altruistic reasons, such as making the data available to the community to foster open science (19.15%), ensuring reproducibility/replicability (11.7%) and transparency (15.96%), supporting teaching (6,38%), and helping colleagues/other researchers (17.02%). However, our respondents also provided other reasons, suggesting that the decision was not just for altruistic reasons. For example, some researchers in our study reported that they benefited from sharing their data, e.g., because it can increase the impact of their research/publication (6.38%) or enable new collaborations (14.89%). The third category of reasons is neither altruistic nor self-serving motives but relates to rules that are followed by data sharing. In such cases, the data were shared due to regulations and requirements, e.g., by institutions (1.06%), funding agencies (1.06%), or journals (5.32%). The main reasons that the respondents did not share their data outside their research team (open question, multiple responses possible, *N* = 153) were that sharing was not considered or needed (30.72%, *n* = 47), legal reasons did not permit sharing (22.22%, *n* = 24), and ethical considerations (13.07%, *n* = 20).

**Figure 2:**
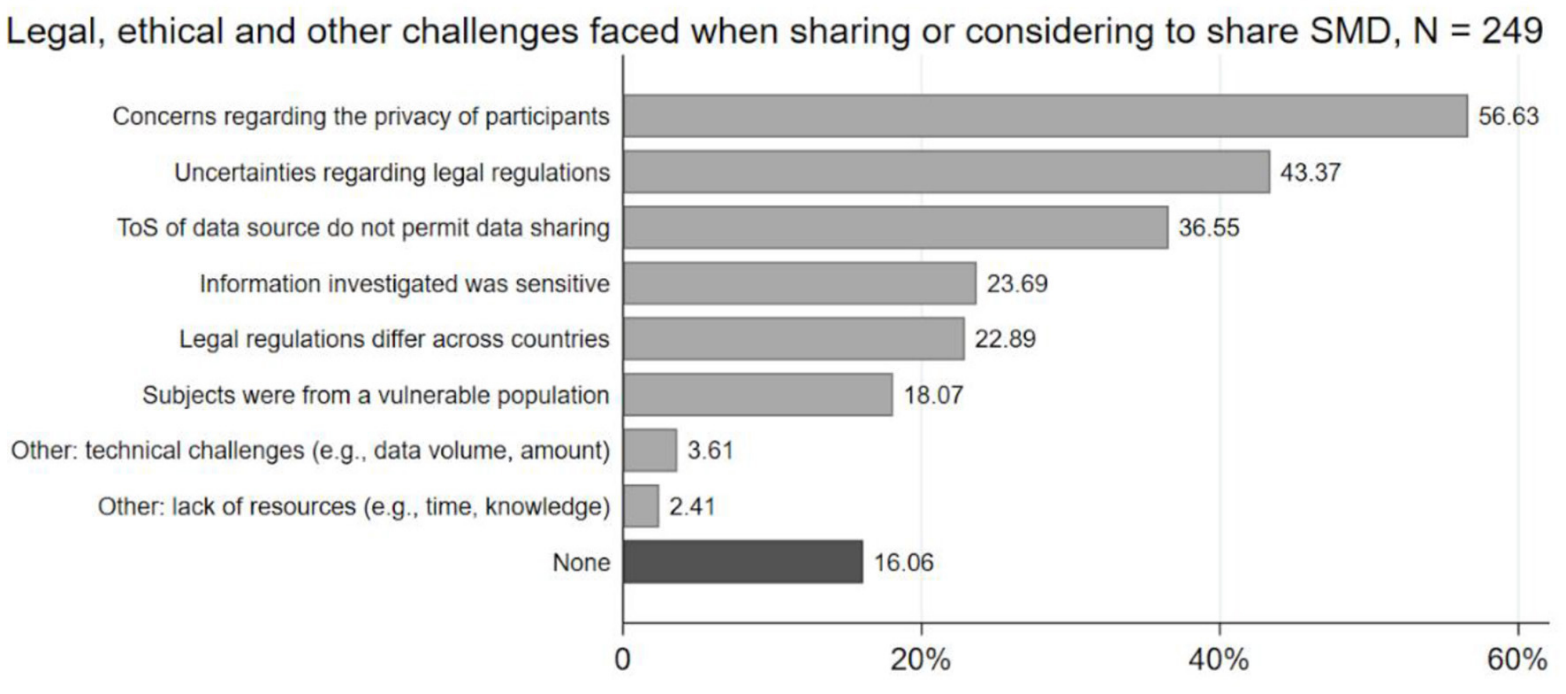
Legal, ethical, and other challenges related to sharing SMD as experienced by researchers.

Respondents were also asked about legal and ethical challenges, particularly when sharing or considering sharing their SMD (multiple responses possible, *N* = 249). Again, we developed an *ad hoc* coding scheme to condense the answers provided to the open-ended questions into coherent categories. The results show (see Figure 2) that participants (*N* = 249) had the strongest concerns about people's privacy (*n* = 141, 56.63%), uncertainties regarding legal regulations (*n* = 106, 43.37%), or the fact that the ToS of the data source did not permit data sharing (*n* = 91, 36.55%).

### 4.2. Predictors of data sharing intentions and differences between sharing types

To assess the differential importance of various predictors of future SMD sharing intentions for different sharing options, we calculated three separate hierarchical (blockwise) linear regression models (one per data sharing type). The outcome variable in the first model was the intention to share SMD publicly (see Table 1). The results show that the demographic control variables (“Female” and “Professorship”) can only explain a very small amount of variance in the outcome variable (1%). Adding past experiences (“used secondary data” and “shared data”) and challenges (“challenge: ToS”, “challenge: legal regulations” and “challenge: people's privacy”) significantly increased the amount of explained variance (21%). In this second step, previous data sharing emerged as a significant positive predictor (b = 1.64, *p* < 0.001). Adding the TPB variables (attitudes: “valuable sharing,” norms: “Expectations of researchers,” and control: “Capacity”) further improved the explanation of variance in the outcome variable (38%). In this third step, in addition to past data sharing experience, the perception of sharing as valuable (b = 0.28, *p* < 0.05), the perceived expectations of other researchers (b = 0.34, *p* < 0.001) and personal capacity (b = 0.24, *p* < 0.01) emerged as significant positive predictors.

**Table 1:** Regression model to predict the intention to share data publicly.

	**(1)**	**(2)**	**(3)**
Female	−0.12 (−0.43)	0.22 (0.90)	0.08 (0.29)
Professorship	0.40 (1.52)	0.19 (0.62)	0.22 (0.98)
Used secondary data		0.51 (1.70)	0.26 (0.87)
Shared data		1.64^***^ (4.60)	0.78^*^ (2.28)
Challenge: ToS		−0.15 (−0.52)	−0.31 (−1.13)
Challenge: legal regulations		0.21 (0.53)	0.15 (0.75)
Challenge: people's privacy		−0.27 (−0.89)	−0.28 (−1.02)
Valuable sharing			0.28^*^ (2.01)
Expectations of researchers			0.34^***^ (3.41)
Capacity			0.24^**^ (2.79)
Autonomy			−0.04 (−0.61)
Constant	3.28^***^ (14.55)	2.50^***^ (7.66)	−0.61 (−0.73)
Observations	177	177	177
*R* ^2^	0.01	0.21	0.38

t statistics in parentheses. Bootstrapped standard errors, listwise solution.

^*^p < 0.05,

^**^p < 0.01,

^***^p < 0.001.

Similar to the first model, in the second model (Table 2) predicting intentions to share SMD under controlled access, demographic controls did not explain a meaningful amount of variance in the first step. However, female gender emerged as a significant positive predictor in step 3 (b = 0.52, *p* < 0.05). In the second block, again, previous data sharing experience was a significant positive predictor (b = 1.05, *p* < 0.001). Unlike for public sharing, however, previous reuse of SMD also turned out to be a significant positive predictor of sharing intentions (b = 0.71, *p* < 0.05). While expectations of other researchers and own capacity were significant predictors in the model for public sharing, this was not the case for the model predicting intentions for sharing under controlled access. The perception of sharing as valuable was a significant positive predictor in step 3 (b = 0.55, *p* < 0.001). As before, adding the TPB variables also significantly increased the amount of explained variance in the outcome variable (from 19% to 35%).

**Table 2:** Regression model to predict the intention to share data under controlled access.

	**(1)**	**(2)**	**(3)**
Female	0.15 (0.63)	0.55 (1.94)	0.52^*^ (2.33)
Professorship	−0.08 (−0.31)	−0.23 (−1.00)	−0.16 (−0.69)
Used secondary data		0.71^*^ (2.53)	0.55 (1.84)
Shared data		1.05^***^ (5.13)	0.35 (1.38)
Challenge: ToS		0.27 (1.07)	0.12 (0.56)
Challenge: legal regulations		0.44 (1.33)	0.34 (1.63)
Challenge: people's privacy		−0.03 (−0.10)	0.03 (0.08)
Valuable sharing			0.55^***^ (4.75)
Expectations of researchers			0.17 (1.49)
Capacity			0.06 (0.63)
Autonomy			0.02 (0.32)
Constant	4.41^***^ (18.91)	3.31^***^ (9.26)	−0.26 (−0.46)
Observations	177	177	177
*R* ^2^	0.00	0.19	0.35

t statistics in parentheses. Bootstrapped standard errors, listwise solution.

^*^p < 0.05, ^**^p < 0.01,

^***^p < 0.001.

Compared to public sharing and sharing under controlled access, the results for the intention to share SMD upon personal request (Table 3) show a somewhat different pattern. While demographic variables have little explanatory power, and previous sharing experiences, again, emerge as a significant predictor in the second block, the significant predictors in the final block are somewhat different compared to the previous models. Here, perceived challenges play a much greater role. Concerns related to platform ToS (b = 0.59, *p* < 0.05) and legal regulations (b = 0.53, *p* < 0.05) were significant positive predictors in this model (step 3). Another positive predictor was perceived autonomy (b = 0.23, *p* < 0.05). Similar to the model for controlled access, the perceived value of sharing also turned out to be a significant positive predictor of the intention to share SMD upon personal request (b = 0.49, *p* < 0.001). As in the other two models, adding the TPB variables greatly increases the amount of explained variance in the outcome variable.

**Table 3:** Regression model to predict the intention to share data upon personal request.

	**(1)**	**(2)**	**(3)**
Female	−0.09 (−0.26)	0.21 (0.80)	0.24 (0.99)
Professorship	0.00 (0.01)	−0.10 (−0.35)	−0.11 (−0.50)
Used secondary data		0.39 (1.33)	0.31 (0.95)
Shared data		0.56 (1.79)	0.09 (0.33)
Challenge: ToS		0.43 (1.40)	0.59^*^ (2.54)
Challenge: legal regulations		0.47 (1.64)	0.53^*^ (2.05)
Challenge: people's privacy		−0.12 (−0.39)	−0.04 (−0.18)
Valuable sharing			0.49^***^ (3.38)
Expectations of researchers			−0.05 (−0.52)
Capacity			0.05 (0.44)
Autonomy			0.23^**^ (3.06)
Constant	4.81^***^ (19.32)	4.04^***^ (12.97)	0.48 (0.59)
Observations	177	177	177
*R* ^2^	0.00	0.10	0.24

t statistics in parentheses. Bootstrapped standard errors, listwise solution.

^*^p < 0.05,

^**^p < 0.01,

^***^p < 0.001.

## 5. Discussion

The findings from our descriptive analyses show that there are various reasons that can motivate researchers or hinder them from sharing SMD. The reasons for sharing SMD can be distinguished into idealistic/altruistic, self-serving, and compliance motives. The analysis identified three key reasons for data sharing: first, researchers' awareness that data sharing is a prerequisite to foster open science, reproducibility, research, and support for others; second, researchers' self-interest in data sharing due to increased research and publication impact and opportunities for collaboration and cooperation with others by sharing their data; and third, stakeholders' requirements that make data sharing obligatory for funding and publications. The first group of reasons can be also subsumed into the category of normative grounds and components of data sharing culture in the academic community (Barbui et al., 2016).

In regard to the reasons that prevent researchers from sharing their data, the obstacles can also be summarized into three categories. First, researchers face legal and/or ethical challenges; in particular, they fear violating peoples' privacy, have uncertainties about legal regulation, or do not have the permission to share the data (by their own institution or by regulations from the ToS of the social media platform). Second, researchers are confronted with a lack of resources, lack of available repositories, or lack of knowledge, especially on technical know-how on data preparation of SMD. Third, researchers do not see a value, benefit, and usefulness in sharing their data, meaning that providing the data to others is not being considered or required thus far. These results show that similar to what has been found for survey data, it is primarily a question of weighing up the costs, efforts, and risks associated with data sharing on the one side and the benefits and potential obligations on the other (Sayogo and Pardo, 2013; Lane et al., 2014; Tenopir et al., 2015; Weller and Kinder-Kurlanda, 2015; Zenk-Möltgen et al., 2018; Van Atteveldt et al., 2019; Hemphill et al., 2021; Thoegersen and Borlund, 2022). Notably, compared to other types of research data, working with SMD entails additional legal risks, such as violating platform ToS. In addition, ensuring the privacy of user data is more difficult compared, e.g., to survey data. The results of the study show that the most prevalent perceived challenges relate to legal questions. This is not surprising given that researchers in the social and behavioral sciences typically do not have extended legal expertise and because there exists a considerable amount of uncertainty in this area, as there is limited case law in many countries and legal regulations also tend to change with technological developments.

In our regression analyses assessing the importance of various types of predictors for future SMD sharing intentions, we see that both past experiences and attitudes, subjective norms, and perceived behavioral control play a role here. In all three models, it appears that past behavior is a significant predictor of future data sharing, proving that researchers who shared their SMD in the past have the intention to share their SMD publicly, under controlled access, or upon personal request. Despite the distinctions we made between categories of predictors and sharing options, one thing that should be considered when interpreting our findings is that some variables were substantially correlated (see the correlation table in Appendix B in Supplementary material), especially the variables assessing the perceived value of sharing, expectations of researchers, capacity, and the intention to share data. This indicates that further distinctions, e.g., regarding different capacities or reasons for valuing data sharing might be useful. On the other hand, our separate data models for the different data sharing types shed light on the differential relevance of the predictor categories.

Taken together, our results show that TPB is a valuable framework for understanding data sharing intentions, as the addition of the associated variables significantly increased the amount of explained variance in the outcome variables in all three models. Norms and perceived behavioral control play a significant role, especially for the question of whether data should be shared publicly without access restrictions. The expectations of other researchers wanting access to the data can also affect researchers' willingness to share SMD. However, public sharing also requires specific capacities. Researchers must be able (i.e., they must have the resources and knowledge) to prepare and anonymize their data in a way that enables them to share them and ensures its reuse value. In the case of sharing the data under controlled access, we found that past reuse experiences and attitudes play a key role in future sharing intentions. Importantly, researchers might have various concerns that do not allow public sharing (e.g., fear of getting scooped, concerns regarding legal regulations, or concerns for people's privacy). We also found differences between the sharing options (public sharing, sharing under access control, and sharing upon personal request) and the relevance of experienced challenges. For example, for sharing upon personal request, perceptions of challenges related to legal questions (including platform ToS) seem to play a larger role. This makes sense as such concerns make researchers more likely to choose this option over public or controlled access sharing. Here, again, a positive attitude toward data sharing and perceiving behavioral control (“Autonomy”) emerged as positive predictors. Notably, many of these capacities are the same for sharing other types of data, but some are specific for SMD (such as knowing and understanding platform ToS). Building and honing the capacities for sharing SMD is an area in which infrastructure institutions can and should offer extended support (e.g., *via* guidelines, consultations, or training). Finding out what specific areas researchers need support for would be an interesting follow-up question from the perspective of research infrastructure institutions.

As with all empirical studies, ours had specific limitations that need to be taken into account when interpreting its results. First, the questions related to the three sharing options were presented consecutively in the survey, so in their answers, people likely compared the option for which they indicated the likelihood of sharing with the previously presented option(s). Another limitation is that our sample of papers is limited to journal publications in well-known databases and, hence, excludes some smaller or newer journals in which other social media researchers may have published empirical papers. Moreover, our sampling approach excluded researchers who have not published in any of the selected journals or have no publications to date, even if they already have experience with SMD in their research. Additionally, participation in our survey was voluntary, so it is possible that researchers who have a positive attitude toward data sharing and who have an interest in or experience in the topic were more likely to participate in the survey (self-selection bias). Finally, as the data we have are cross-sectional in nature and there may be other relevant predictors that we did not consider in our study (e.g., differences between platform or SMD formats), we cannot make causal claims about the relationship between the attributes, experiences, and attitudes of researchers and SMD sharing behavior. Besides, we focused on individual-level factors. Factors that exist on a systemic level, such as data sharing cultures in specific disciplines or fields are also relevant for SMD sharing. Assessing differences in data sharing cultures and their impact on individual decisions and intentions, however, requires data that allows for systematic comparisons between disciplines or fields of study. This would be an interesting avenue for future research.

Despite the strengths associated with a systematic quantitative approach of investigating researcher's SMD sharing intentions *via* an online survey, our study has also some methodological as well as content-related limitations. One of them is that SMD sharing decisions and intentions were investigated in general terms—independent of the specific social media platforms and data formats. Distinguishing between origins and formats of SMD could result in a more detailed picture of respondents' intentions to share data. Another limitation is that we did not compare SMD sharing intentions across different disciplines. One of the reasons is that in our study, 38% of the respondents worked more than in one discipline area. The interdisciplinary environment does not provide a ground for a direct comparison of disciplines. However, given different data sharing practices and cultures across disciplines, such a comparison might be a worthwhile endeavor for future research. Regarding the methodology, the open-ended responses in our survey can only provide limited in-depth insights. Obtaining more in-depth insights into an individual's experiences, attitudes, and intentions requires qualitative methods as employed in the studies by Weller and Kinder-Kurlanda (2015). As a final remark, the survey was conducted during the COVID-19 pandemic, but we did not investigate the effect of the pandemic on data sharing intentions. As open data were an important element in understanding and dealing with the pandemic, this may well have an effect on the data sharing attitudes and intentions of social media researchers as well.

## 6. Conclusion

The results of our study have extended the findings of previous research on data sharing in general and working with SMD in particular. The insights gained here can serve to inform guidelines and services provided by various stakeholders. Notably, the role of predictors varies across different modes of intentions to share SMD—publicly, under controlled access or upon personal request. Above all, the results of the study showed that researchers would more likely share their data (a) if they have already shared their data in the past (for sharing publicly and under controlled access), (b) if there is some external pressure and expectations (for sharing publicly), (c) if they have a positive opinion of data sharing and recognize the value of the data for reuse (for all modes of sharing), and (d) if they fell capable (for public sharing) of and autonomous (for sharing upon personal request) with respect to sharing their data. While previous research has typically focused on one particular type of data (e.g., survey data or SMD), ours distinction between data sharing modes allows for this assessment of the differential relevance of predictors. Employing TPB as the theoretical framework allowed us to investigate different types of potential influencing factors on the individual level and to assess general attitudes and intentions on a more general level (i.e., not tied to one particular use case).

Stakeholders who could and should be particularly interested in these results and researchers' experiences, challenges, and opinions in dealing with SMD are the institutes and universities where the researchers are trained and employed and the archives and repositories, funding agencies, and journal owners. Institutions and universities could, e.g., improve the capabilities of researchers by providing resources (time, technical support, legal counseling), providing training, and including data management in their educational curricula. The findings from our study are also of interest to data archives. One thing that can be concluded from our findings in that regard is that researchers likely need more extensive advice and more intensive support for archiving SMD. For example, researchers could benefit from archival staff with legal expertise or documents, including legal and ethical advice/recommendations that archives could develop. In addition, archives could also partake in the training of researchers in regard to the preparation and documentation of SMD. The third group of stakeholders for whom our findings can be of value are journals and publishers. Providing clear guidance as well as procedures for sharing data (including SMD) and explicit data sharing policies could be steps that this group of stakeholders could take to increase SMD sharing. Similar conclusions can be drawn for funders. For this group, providing special/extra funding for research data management as well as research data infrastructures and their development could be helpful steps for facilitating and fostering SMD sharing.

To extend our findings, future research could engage in more in-depth analyses by extending the set of predictors, including other perspectives in addition to the researchers' individual characteristics and past behavior. For example, disciplinary data sharing practices and cultures likely play a role for individual decisions and the (perceived) role of archives and repositories as a supporting system for researchers in enabling data sharing needs further investigation. Regarding the role of archives, one follow-up question could be, what the roles of perceived control factors, such as the availability of and support by repositories and archives, are for predicting researchers' decisions to share or not to share their SMD. Due to the insecurities identified in our survey study that arise when researchers are faced with legal and ethical questions when sharing SMD, it would be interesting to gain insights into the potential of archives and repositories as support structures and facilitators of data sharing. Given their purpose, archives have an interest in receiving SMD, and educating and supporting researchers in documenting and managing them are means to achieve this. In addition, a more comprehensive study could be conducted by including other types of new data (such as digital trace data more broadly) or by defining the term “data” or “data format” in a more detailed way (e.g., distinguishing between unstructured and structured data, such as data in the classical “rectangular format” of survey data).

Another promising area for future research is to advance the implication of TPB for predicting intentions to share SMD. This study used only one direct measure for the main concepts related to TPB and did not cover the potential manifold relationships of attitudes, social norms, capacity and autonomy. A study of indirect indicators can shed further light on the relative weights of the components that make up these concepts in predicting intentions. Eventually, extending research on social media data sharing following these future methodological trajectories can result in more precise recommendations and guidance for archives and repositories.

In summary, if stakeholders support (to overcome legal and ethical hurdles), require (to motivate researchers/institutions to invest resources), and promote (to raise the awareness that the data are valuable) data sharing, sharing SMD could become more common. In this process, however, it is crucial to not only take into account and address the specific attributes of SMD but also the experiences, attitudes, norms, and perceived behavioral control among researchers who collect and work with SMD.

## Data availability statement

The original contributions presented in the study are included in the article/Supplementary material, and available via this link: https://doi.org/10.7802/2418 further inquiries can be directed to the corresponding author.

## Author contributions

The authors confirm contribution to the paper as follows: study conception and design: EA, KB, and JB. Data collection, analysis and interpretation of results, and draft manuscript preparation: EA, KB, JB, and YV. All authors reviewed the results and approved the final version of the manuscript.

## Footnotes

^1^In this paper, SMD are defined as any data that have been retrieved from social media sites and applications in which users generate and share content (e.g., tweets, Instagram photos/videos, Reddit comments, etc.) which were collected by scientists for their research or have been used for secondary analysis.

^2^By *data reuse*, we mean the use of data for a research activity or purposes other than those for which it was originally intended/collected. This can, e.g., be done by downloading data from data repositories or by requesting data *via* personal communication. In this case, the researcher who accesses these already collected data is the secondary data user.

^3^For the purposes of this paper, *data sharing* is defined as the act of releasing data in a form that can be used by other researchers. This can, e.g., be done through private exchange, public repositories, or other forms of data provision (Pasquetto et al., 2017).

^4^See https://www.go-fair.org/fair-principles/.

^5^The order of reasons is random and not an indication of frequency or importance.

^6^See https://mjl.clarivate.com/search-results.

^7^The list of journals will be kept confidential to avoid jeopardizing the privacy of the participants.

^8^Please note that our sample was probably biased by the interest in topic data sharing and voluntary participation (without any reward). Thus, the absolute values of the measurements are quite possibly not representative of this scientific community as a whole. Additionally, the group is difficult to define due to people's different disciplines and SMD being a heterogeneous concept. However, for the relative association (as represented by the regression coefficients in our quantitative analysis) between data sharing intentions and the predictors we considered, this systematic bias should not matter.

^9^The wording of the question items that were used for this paper can be found in Appendix A in Supplementary material.

^10^The figure includes only those categories for which at least 1% of the total respondents reported having faced a challenge.
