# Supplementary material for: Sharing social media data: The role of past experiences, attitudes, norms, and perceived behavioral control
Source: Front Big Data. 2023 Jan 16;5:971974. doi: 10.3389/fdata.2022.971974 (PMC9885192; doi:10.3389/fdata.2022.971974)
Supplement: Supplementary file 1 [file Table_1.DOCX]

**Appendix A – Question Items**

Question Items used in the study

| **Construct** | **Items** | **Item example** | **Scale** |
| --- | --- | --- | --- |
| Professorship | 1 | What is your current occupational status? | Please check all that apply. |
|  |  |  |  |
| Gender | 1 | To which gender identity do you most identify? |  |
|  |  |  |  |
| Use of primary/secondary data | 1 | When working with social media data, do you or your collaborators collect the social media data yourself (primary data) or retrieve it from data repositories, archives, or other existing collections (secondary data)? | Please check all that apply. |
| Intention | 3 | How likely or unlikely is it that you will share social media data with others outside your research team within the next 3 years publicly (open with no restrictions)? | 1 = extremely unlikely  7 = extremely likely |
| Attitudes | 2 | Generally speaking, sharing social media data with others outside of your research team within the next 3 years, would be | 1 = extremely worthless  7 = extremely valuable |
| Subjective norms | 2 | Please indicate how much you agree or disagree with the following statements:  Most researchers in my scientific discipline expect me to share my social media data with others outside of my research team within the next 3 years. | 1 = strongly disagree  7 = strongly agree |
| Perceived behavioral control | 2 | Please indicate how much you agree or disagree with the following statements:  I am confident that, if I wanted to, I would be capable of sharing my social media data with others outside of my research team within the next 3 years. | 1 = extremely unlikely  7 = extremely likely |
| Past behavior: Reasoning (Legal and ethical challenges) | 2 | Which of the following legal or ethical challenges have you faced when sharing or considering to share social media data with others outside of your research team?   - The Terms of Service of the data source do not permit sharing the data - Legal regulations differ across countries in collaborative projects - Uncertainties regarding legal regulations - The information investigated was sensitive - The research subjects were from a vulnerable population - Concerns regarding the privacy of participants - Other (please specify and separate multiple answers with a semicolon): ______________ - None - *Don’t know* | Please check all that apply. |
|  |  |  |  |

**Appendix B – Additional Tables**

Demographics of the survey respondents

| Demographic Variables | Total | % |
| --- | --- | --- |
|  |  |  |
| *Gender* |  |  |
| Male | 128 | 51.41 |
| Female | 109 | 43.78 |
| Missing | 12 | 4.82 |
|  |  |  |
| *Year of birth* |  |  |
| 1991-1995 | 41 | 16.47 |
| 1986-1990 | 50 | 20.08 |
| 1981-1985 | 56 | 22.49 |
| 1976-1980 | 30 | 12.05 |
| 1971-1975 | 29 | 11.65 |
| <=1970 | 19 | 7.63 |
| Missing | 24 | 9.64 |
|  |  |  |
| *Research area** |  |  |
| Anthropology | 9 | 3.61 |
| Communication sciences | 164 | 65.86 |
| Computer science | 34 | 13.65 |
| Demography | 2 | 0.80 |
| Economics | 5 | 2.01 |
| History | 4 | 1.61 |
| Human geography | 5 | 2.01 |
| Linguistics | 15 | 6.02 |
| Political science | 49 | 19.68 |
| Psychology | 24 | 9.64 |
| Social work | 1 | 0.40 |
| Sociology | 61 | 24.50 |
| Other | 45 | 18.07 |
|  |  |  |
| *Affiliation** |  |  |
| University, college, or technical university | 227 | 91.16 |
| Other | 31 | 12.45 |
|  |  |  |
| *Occupation** |  |  |
| Student/Doctoral/PhD/Researcher (without doctorate/PhD) | 49 | 19.68 |
| Researcher (with doctorate/PhD)/Post-Doc/Lecturer | 73 | 29.32 |
| Professor/Assistant professor/Associate professor | 127 | 51.00 |
|  |  |  |
| Total | 249 | 100 |
| * Multiple answers possible |  |  |

What were the reasons for sharing your social media data with others outside of your research team? [open question, ad hoc coding]

|  | **N** | **%** |
| --- | --- | --- |
| to help colleagues/other researchers | 94 | 17.02 |
| increase impact of research | 94 | 6.38 |
| foster Open Science/Access | 94 | 19.15 |
| data has been requested/asked for by others | 94 | 10.64 |
| teaching matters/educational reasons | 94 | 6.38 |
| promote research/advancement of field | 94 | 10.64 |
| collaboration/cooperation | 94 | 14.89 |
| conference presentation | 94 | 1.06 |
| to ensure reproducibility/replicability | 94 | 11.70 |
| data used for bartering | 94 | 1.06 |
| publication requirements | 94 | 5.32 |
| transparency of research findings | 94 | 15.96 |
| requirement of funding agency | 94 | 1.06 |
| curiosity | 94 | 1.06 |
| moral obligation | 94 | 2.13 |
| requirement of institution | 94 | 1.06 |

What were the reasons for not sharing your social media data outside of your research team so far? [open question, ad hoc coding]

|  | **N** | **%** |
| --- | --- | --- |
| lack of cooperation | 153 | 2.61 |
| sharing not considered/needed | 153 | 30.72 |
| legal reasons | 153 | 22.22 |
| data not useable/reusable/relevant | 153 | 5.23 |
| no suitable archive/repository found | 153 | 0.65 |
| lack of know-how/information | 153 | 3.27 |
| ethical reasons (e.g., informed consent) | 153 | 13.07 |
| not allowed to share | 153 | 7.84 |
| restrictions of institution | 153 | 3.92 |
| lack of resources | 153 | 2.61 |
| lack of incentives | 153 | 0.65 |

How likely or unlikely is it that you will share social media data with others outside your research team within the next 3 years [1-7]

|  | **N** | **mean** | **sd** |
| --- | --- | --- | --- |
| intention to share publicly | 229 | 3.28 | 2.07 |
| intention to share under controlled access | 230 | 4.33 | 1.79 |
| intention to share only upon personal request | 229 | 4.67 | 1.80 |

Attitudes toward data sharing: worthless-valuable to share SMD [1-7]

|  | **N** | **mean** | **sd** |
| --- | --- | --- | --- |
| worthless-valuable to share SMD | 222 | 5.46 | 1.09 |

Subjective norms. Please indicate how much you agree or disagree with the following statements
[1-7]

|  | **N** | **mean** | **sd** |
| --- | --- | --- | --- |
| expectation of researchers in scientific discipline | 217 | 3.47 | 1.70 |

Perceived behavioral control. Please indicate how much you agree or disagree with the following statements [1-7]

|  | **N** | **mean** | **sd** |
| --- | --- | --- | --- |
| capability to share SMD | 231 | 4.84 | 1.65 |
| autonomy to share SMD | 235 | 4.31 | 2.06 |

Correlation matrix

|  | (1) | (2) | (3) | (4) | (5) | (6) | (7) | (8) | (9) | (10) | (11) | (12) | (13) | (14) |
| --- | --- | --- | --- | --- | --- | --- | --- | --- | --- | --- | --- | --- | --- | --- |
| (1) Intention to share publicly | 1.00 |  |  |  |  |  |  |  |  |  |  |  |  |  |
| (2) Intention to share controlled | 0.37^***^ | 1.00 |  |  |  |  |  |  |  |  |  |  |  |  |
| (3) Intention to share personal request | 0.17^*^ | 0.55^***^ | 1.00 |  |  |  |  |  |  |  |  |  |  |  |
| (4) Female | -0.04 | 0.04 | -0.02 | 1.00 |  |  |  |  |  |  |  |  |  |  |
| (5) Professorship | 0.10 | -0.03 | 0.00 | -0.08 | 1.00 |  |  |  |  |  |  |  |  |  |
| (6) Used secondary data | 0.23^**^ | 0.27^***^ | 0.18^*^ | -0.21^**^ | 0.02 | 1.00 |  |  |  |  |  |  |  |  |
| (7) Shared data | 0.43^***^ | 0.34^***^ | 0.21^**^ | -0.17^*^ | 0.14 | 0.31^***^ | 1.00 |  |  |  |  |  |  |  |
| (8) Challenge: ToS | 0.03 | 0.14 | 0.17^*^ | -0.22^**^ | 0.05 | 0.16^*^ | 0.16^*^ | 1.00 |  |  |  |  |  |  |
| (9) Challenge: legal regulations | 0.07 | 0.17^*^ | 0.17^*^ | -0.08 | 0.03 | 0.08 | 0.11 | 0.23^**^ | 1.00 |  |  |  |  |  |
| (10) Challenge: people's privacy | -0.08 | 0.02 | 0.02 | -0.07 | -0.04 | -0.09 | -0.01 | 0.25^***^ | 0.29^***^ | 1.00 |  |  |  |  |
| (11) Valuable sharing | 0.41^***^ | 0.50^***^ | 0.36^***^ | -0.13 | -0.03 | 0.19^*^ | 0.39^***^ | 0.19^*^ | 0.14 | -0.02 | 1.00 |  |  |  |
| (12) Expectations of researchers | 0.49^***^ | 0.41^***^ | 0.17^*^ | 0.03 | 0.10 | 0.22^**^ | 0.35^***^ | 0.16^*^ | 0.13 | 0.07 | 0.43^***^ | 1.00 |  |  |
| (13) Capacity | 0.43^***^ | 0.29^***^ | 0.22^**^ | -0.05 | 0.01 | 0.18^*^ | 0.38^***^ | -0.04 | -0.08 | -0.08 | 0.33^***^ | 0.41^***^ | 1.00 |  |
| (14) Autonomy | 0.05 | 0.00 | 0.19^*^ | 0.08 | 0.13 | 0.01 | 0.04 | -0.29^***^ | -0.18^*^ | -0.10 | -0.08 | -0.01 | 0.35^***^ | 1.00 |
| - *N* | 177 |  |  |  |  |  |  |  |  |  |  |  |  |  |

^*^ *p* < 0.05, ^**^ *p* < 0.01, ^***^ *p* < 0.001
